# Organizing Pneumonia Following Recombinant Zoster Vaccination: A Rare Adverse Reaction

**DOI:** 10.7759/cureus.21181

**Published:** 2022-01-12

**Authors:** Imran Khawaja, Mohammed Al-Ourani

**Affiliations:** 1 Pulmonary Medicine, Marshall University Joan C. Edwards School of Medicine, Huntington, USA; 2 Pulmonary and Critical Care Medicine, Marshall University Joan C. Edwards School of Medicine, Huntington, USA

**Keywords:** adverse effects, vaccination, vaccine, shingles, zoster, organizing pneumonia

## Abstract

We describe a case of a severe but rare adverse reaction to recombinant varicella-zoster virus (VZV) vaccination: a 67-year-old female admitted with gradual onset of shortness of breath, hypoxia, and fever following VZV vaccination. The clinical picture and radiologic presentation mimicked COVID-19 pneumonia. However, repeated testing for COVID-19 by PCR was negative. A diagnosis of organizing pneumonia was made on transbronchial biopsies. The patient responded well to steroids and improved both clinically and radiographically. This case illustrates not only a rare and unreported complication from vaccination but also raises the awareness during the COVID-19 pandemic that other etiologies can mimic COVID-19 pneumonia. Physicians should be aware of other diagnoses that can mimic COVID-19 infection.

## Introduction

Herpes zoster (shingles) is a common sequela of the reactivation of varicella in older adults. It is recommended in adults above 50 years of age to reduce the risk of developing shingles by 90% [1]. Most of the side effects are mild at the injection site or systemic, such as mild fever or fatigue within seven days of vaccination [2]. Organizing pneumonia is an inflammatory response that can be idiopathic or secondary to exposure to any allergies, infections, inflammation, or connective tissue disorders. We present a rare case of a patient who developed hypoxia and pulmonary infiltrates shortly after receiving her first dose of the varicella-zoster virus (VZV) vaccine.

## Case presentation

A 67-year-old female presented to the hospital with shortness of breath of over two to three prior to hospitalization. Her chest X-ray and CT scans in the emergency room showed bilateral patchy peripheral infiltrates. Her past medical history is significant for coronary artery disease, hypertension, and hyperlipidemia. Her surgical history is significant for diverticulitis status post partial colectomy and status post hysterectomy.

The patient had COVID-19 about eight months ago prior to this presentation. She had no pulmonary symptoms at that time. She did not require any hospitalization and recovered fully. She was fully vaccinated with the Moderna vaccine four months prior to the presentation. She received shingles vaccination about four weeks prior to presentation. The patient stated that, since receiving her VZV vaccine, she started getting progressive shortness of breath and had constitutional symptoms with low-grade fever.

The patient smoked for about 15 years but quit about six months prior to presentation. Her home medications included aspirin, citalopram, iron, hydrochlorothiazide, Synthroid, multivitamin, olmesartan (Benicar), bupropion (Wellbutrin XL), icosapent ethyl (Vascepa), and Align (probiotic).

**Figure 1:**
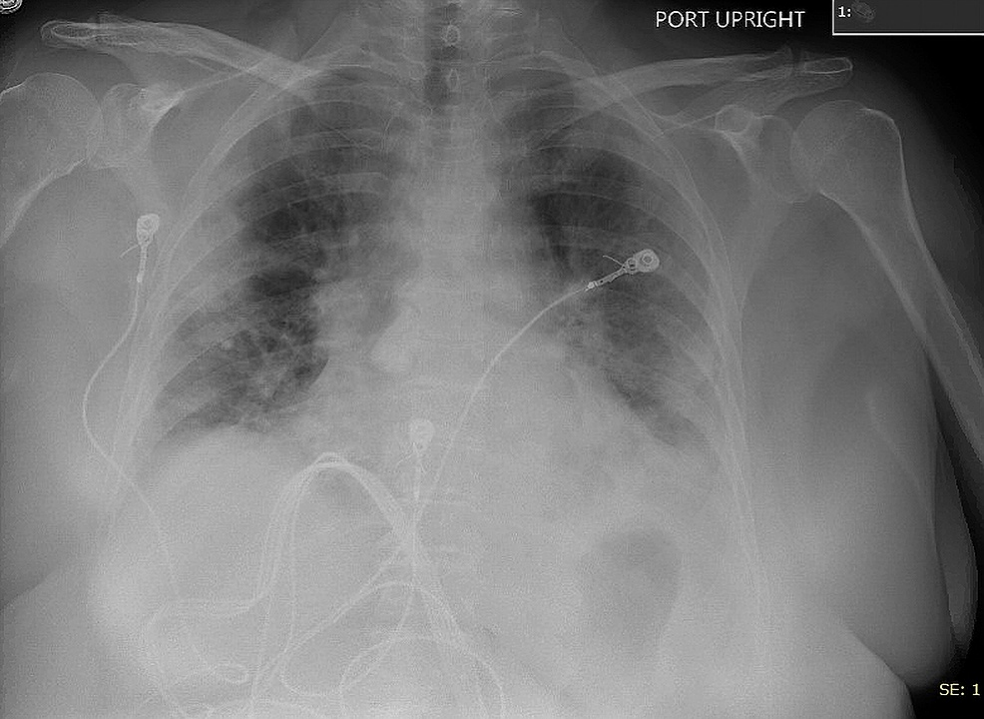
Admission chest X-ray showing bilateral peripheral infiltrates

**Figure 2:**
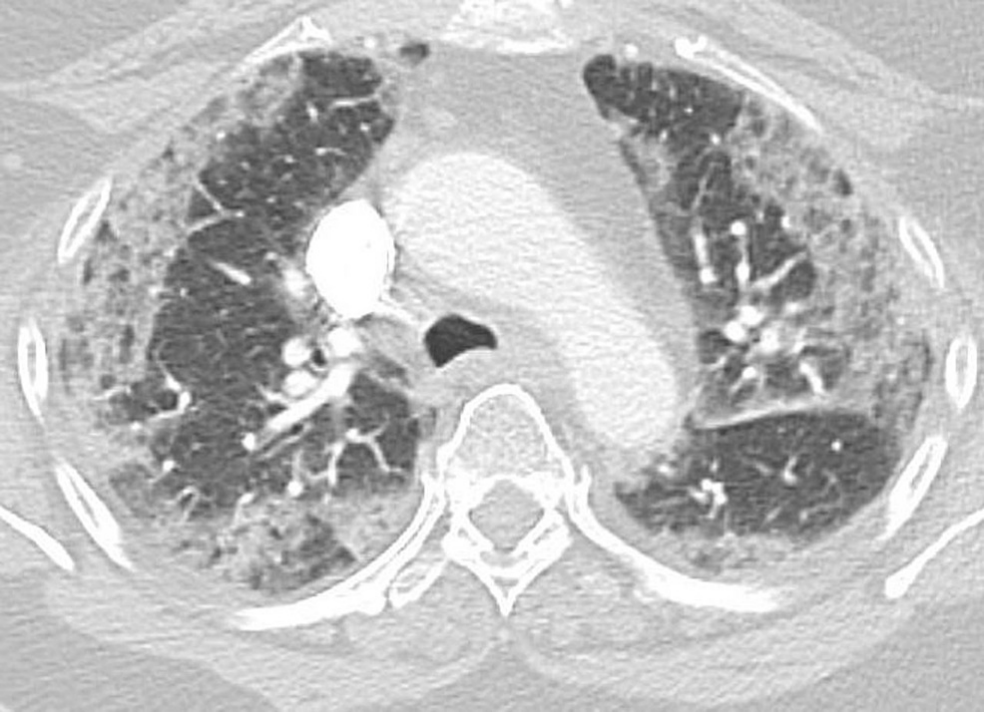
Admission chest CT scan showing bilateral peripheral ground-glass opacities

On physical examination, her temperature was 35.9°C, and her oxygen saturation was 93%. Other vital signs were normal. The physical examination was normal. Her lungs were clear to auscultation, and there was no evidence of any skin rashes. Laboratory data included a WBC count of 14,800, 88% neutrophils, 4% bands, 6% lymphocytes, and 5.8% eosinophils. Hemoglobin and hematocrit were 11.1 g/dL and 30.6%, respectively. Her platelet count was 542,000, lactic acid was 0.5, and procalcitonin level was less than 0.05. Her CRP was less than 5. Her nasopharyngeal swab for COVID- PCR test on two occasions was negative. Her sedimentation rate was 80. Rheumatoid factor was less than 10. ANA was slightly positive at 40, which was nonspecific. The IgE level was 5.5. Her arterial blood gas showed a pH of 7.51 with pCO_2_ of 31, PaO_2_ of 51, and O_2_ saturation of 86% on room air. COVID-19 antibody titers were positive at 106 AU/mL due to a prior COVID-19 infection eight months ago. Her chest X-ray and CT scans showed bilateral peripheral ground-glass infiltrates (Figure 1 and Figure 2).

**Figure 3:**
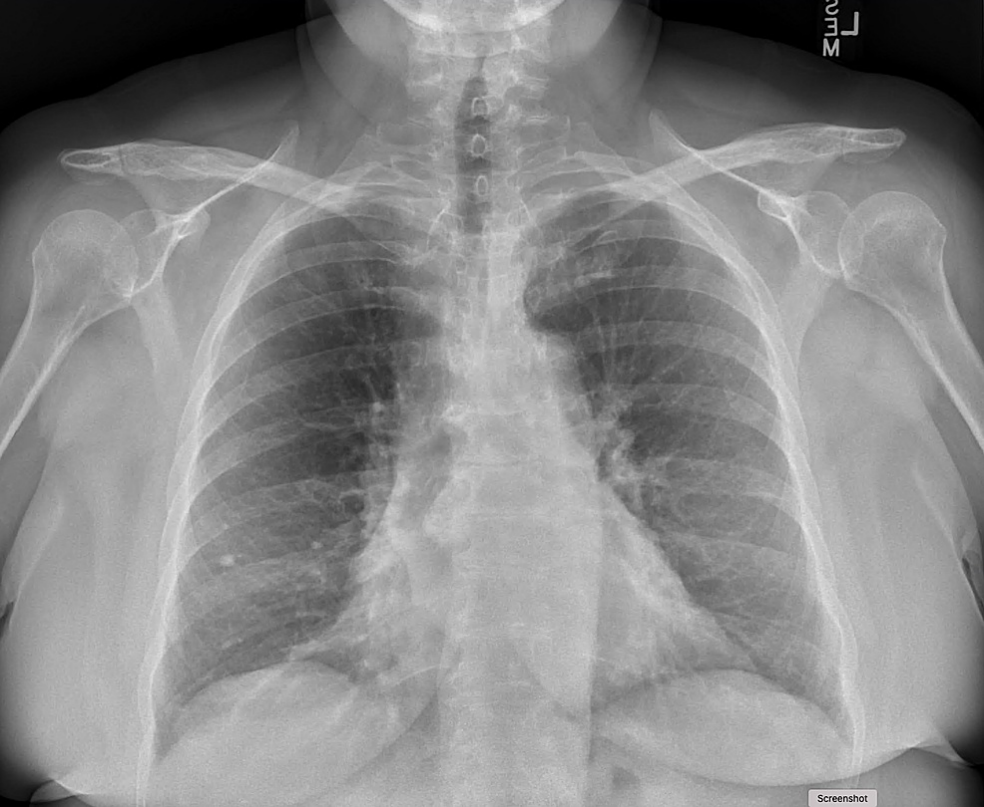
Chest X-ray following therapy showing complete clearing of bilateral infiltrates

**Figure 4:**
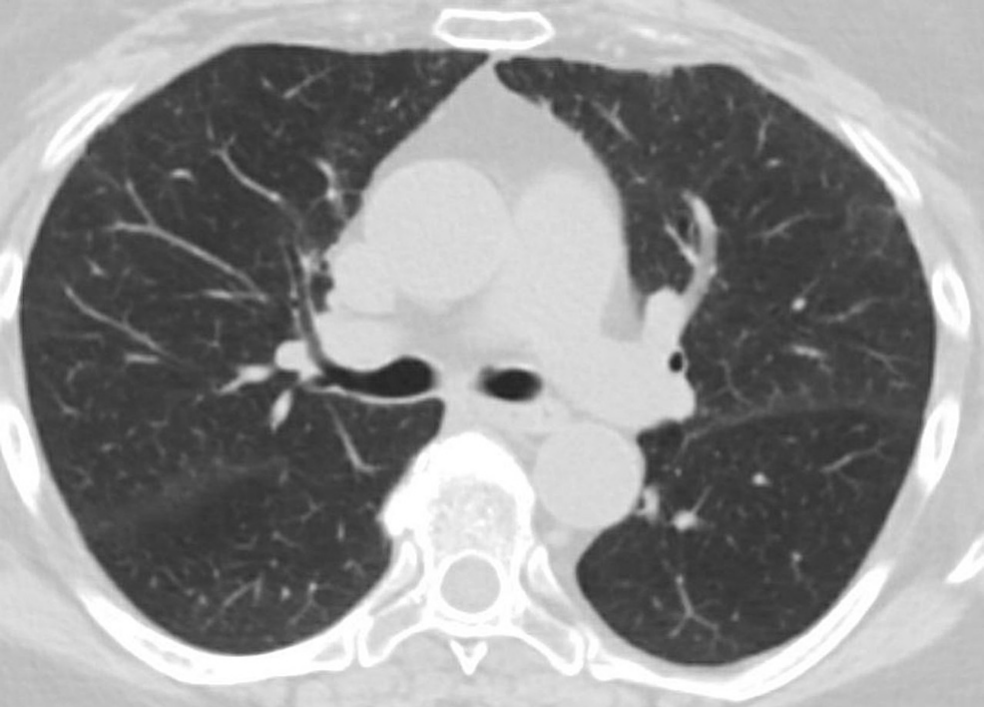
Chest CT scan showing complete clearing of pulmonary opacities after 12 weeks of treatment

The differential diagnosis included acute or chronic eosinophil pneumonia, cryptogenic organizing pneumonia (COP), COVID-19-related acute or chronic interstitial pneumonitis, chronic interstitial lung disease, and hypersensitivity pneumonitis. The patient underwent bronchoscopy with bronchoalveolar lavage (BAL) and trans-bronchial biopsies (TBBx). BAL showed 3% epithelial cells, 40% macrophages, 40% neutrophils, 0% lymphocytes, and only 12% eosinophils. TBBx showed acute and chronic inflammation as well as alveolar spaces filled with young fibroblast consistent with organizing pneumonia. The findings were consistent with acute organizing pneumonia. GMS and PAS stains were negative for any fungi. AFB stains were also negative. Cytology was negative for any malignant cells. A diagnosis of acute organizing pneumonia due to the varicella-zoster virus vaccine was made based on the temporal relationship, laboratory data, and bronchoscopy findings. The patient was started on oral prednisone. A follow-up CT scan after 12 weeks of prednisone therapy showed complete resolution of bilateral interstitial pneumonitis (Figure 3 and Figure 4).

## Discussion

Our patient is a 67-year-old female with no known prior lung disease who presented with progressive onset of shortness of breath for about two to three weeks in duration. Her chest X-ray and CT scan showed bilateral pulmonary infiltrates with ground-glass appearance and peripheral distribution.

The patient underwent extensive evaluation. Nasopharyngeal swab for COVID-19 by PCR was negative twice. Her CRP level was also normal. Given the negative COVID-19 PCR at the time of presentation as well as normal CRP levels, it was highly unlikely that she had acute COVID-19 pneumonia. Post-COVID-19 interstitial lung disease was also felt unlikely especially since her current illness started eight months after she had the COVID-19. She did not have any pulmonary symptoms at the time of her COVID-19 infection. The patient denied any history of exposure to any chemicals, irritants, or any other noxious stimulus to suggest any form of hypersensitivity pneumonitis. The possibility of acute versus chronic eosinophilic pneumonia was also raised given the 5.8% eosinophils in the peripheral blood. However, BAL only showed 12% eosinophils. Eosinophils greater than 25% in BAL are required for the diagnosis of acute or chronic eosinophilic pneumonia [3]. TBBx did not show any eosinophilic infiltration or granulomas to suggest eosinophilic pneumonia or hypersensitivity pneumonitis. GMS and PAS stains were negative for any fungi. AFB stains were also negative. Cytology was negative for any malignant. TBBx showed acute and chronic inflammations as well as alveolar spaces filled with young fibroblasts. These findings were highly consistent with a diagnosis of acute organizing pneumonia. Organizing pneumonia was first described by Beasley et al. [4], where the lung parenchyma has intra-alveolar fibrin in the form of fibrin "balls" within the alveolar spaces. This histological pattern has been described as acute fibrinous and organizing pneumonia (AFOP). This histological pattern is different than seen in diffuse alveolar damage (DAD) or cryptogenic organizing pneumonia (COP).

Recombinant zoster vaccine (RZV) is designed to restore both cell- and humoral-mediated immunity. The formulation is a combination of glycoprotein E (gE) along with a liposomal-based adjuvant called AS01. This combination consistently enhances T-cell responses in addition to antibody production [5]. The most common side effects include injection site reactions such as pain, redness, and swelling. Systemic side effects include myalgia, fever, headache, shivering, fatigue, and gastrointestinal symptoms. A 10-year review of post-marketing safety experience showed that 93% of adverse reactions were nonserious. Injection site reactions were the most reported adverse experience. Most of the adverse reactions were reported within two weeks of vaccination [6]. Similarly, another study looking at the safety of zoster vaccine from a large managed care cohort found zoster vaccine to be safe, with a small increased risk of allergic reactions one to seven days after vaccination [7]. The exact mechanism of action for acute lung injury following VZV vaccination is not known. We believe that it may be due to an exaggerated immune response. Such an autoimmune/inflammatory syndrome induced by the adjuvants (ASIA) has been reported [8]. Adjuvants themselves do not mount an immune response, but they help in the production of an intense response against inoculated antigens. This helps in reducing frequency and dosing to attain acquired preventive immunity. The adjuvant used in VZV vaccination is AS01, which could have caused an exaggerated immune response, causing an acute lung injury that leads to organizing pneumonia.

## Conclusions

Recombinant zoster vaccine (RZV) is generally safe and >90% effective in preventing the reactivation of herpes zoster in older age. The majority of the side effects are mild at the site of local injection or systemic, such as low-grade fever, myalgias, or fatigue. These side effects normally abate by seven days. Secondary organizing pneumonia as described in our case report is the first serious pulmonary side effect of vaccination. Onset was insidious with symptoms starting within one to two weeks of vaccination and progressing over the next three to four weeks. The pathology findings revealed organizing pneumonia, and symptoms completely resolved after treatment. It is felt that this could be a hyperimmune response to the adjuvants used in the vaccine.
